# Emerging Pro-neurogenic Therapeutic Strategies for Neurodegenerative Diseases: A Review of Pre-clinical and Clinical Research

**DOI:** 10.1007/s12035-024-04246-w

**Published:** 2024-05-31

**Authors:** Mariana Vassal, Filipa Martins, Bruno Monteiro, Simone Tambaro, Ricardo Martinez-Murillo, Sandra Rebelo

**Affiliations:** 1 Department of Medical Sciences, Institute of Biomedicine (iBiMED), University of Aveiro, Aveiro, Portugal; 2 Department of Neurobiology, Care Sciences and Society, Division of Neurogeriatrics, Karolinska Institutet, Huddinge, Sweden; 3 Neurovascular Research Group, Department of Translational Neurobiology, Cajal Institute (CSIC), Madrid, Spain

**Keywords:** Adult neurogenesis, Neurodegenerative diseases, Neural stem cells, Therapeutics, Pre-clinical studies, Clinical research

## Abstract

The neuroscience community has largely accepted the notion that functional neurons can be generated from neural stem cells in the adult brain, especially in two brain regions: the subventricular zone of the lateral ventricles and the subgranular zone in the dentate gyrus of the hippocampus. However, impaired neurogenesis has been observed in some neurodegenerative diseases, particularly in Alzheimer’s, Parkinson’s, and Huntington’s diseases, and also in Lewy Body dementia. Therefore, restoration of neurogenic function in neurodegenerative diseases emerges as a potential therapeutic strategy to counteract, or at least delay, disease progression. Considering this, the present study summarizes the different neuronal niches, provides a collection of the therapeutic potential of different pro-neurogenic strategies in pre-clinical and clinical research, providing details about their possible modes of action, to guide future research and clinical practice.

## Introduction

For decades, it has been known that terminally differentiated neurons are incapable of re-entering the cell division cycle and undergo mitosis [1]; however, new neurons can be generated through the differentiation of neural precursors such as neural stem and progenitor cells, in a process called neurogenesis [2]. Neurogenesis was conventionally perceived to occur only during the embryonic and pre-natal stages in mammals [3] and even though it is most active during embryonic development, accumulating evidence has repeatedly shown that adult mammals (including the human adult brain) maintain this capacity throughout their life [4–6], although limited to specific events and restricted regions [7]. It starts with the proliferation of precursor cells which then commit to a neuronal phenotype and become neuroblasts that migrate and differentiate into mature neurons. Finally, these mature neurons become functionally integrated into pre-existing neural networks [8]. Adult neurogenesis has been best described in the subgranular zone (SGZ) of the dentate gyrus (DG) of the hippocampus [9], and in the subventricular zone (SVZ) adjacent to the lateral ventricles [10]. In the SGZ, neurogenesis is responsible for the control of spatial learning and memory, and for the integration of cognition and emotion [11], whereas in the SVZ, the newly generated neurons migrate to the olfactory bulb (OB) [12] to participate in the control of olfactory inputs [11]. However, multiple additional brain areas have been reported as having some adult neurogenic activity, namely the hypothalamus [13, 14], striatum [15, 16], *substantia nigra* [17, 18], cortex [19–21], and amygdala [22]. The discovery of these locations has refuted the idea that the adult human brain is an immutable structure, but rather an organ that possesses a certain level of plasticity that can alter its cellular composition by producing new neurons that are integrated into existing neuronal circuits [23].

These findings had a significant impact on the understanding of neurodegenerative disorders. For example, in conditions such as Alzheimer’s disease (AD), it is believed that a reduction in neurogenesis may contribute to the decline in cognitive function associated with the disease [24, 25]. Importantly, this loss has been reported to occur prior to the deposition of amyloid-β plaques, which is one of the pathological hallmarks of AD [26]. Therefore, there is a compelling need to develop pro-neurogenic therapies, since they could have a significant impact on improving brain function and overall quality of patients’ life [27]. Most of the ongoing research is mainly directed toward promoting endogenous neurogenesis, which involves the manipulation of endogenous mechanisms of the adult brain to repair itself [28, 29]. However, this approach is only effective during the early stages of neurodegenerative diseases [30]. During the advanced stages of these diseases, the damage is typically too extensive for the brain’s innate neurogenic capacity to fully restore its function [25]. In such cases, exogenous therapies can induce neurogenesis by introducing new cell populations into the damaged tissues [31]. Therefore, this review aims to provide a comprehensive overview of the therapeutic potential of these different strategies in pre-clinical studies and clinical research. The review also includes discussions on the most probable modes of action of each strategy, strengths, limitations, and gaps in knowledge to guide future research and clinical practice.

## Neurogenic Niches in the Adult Brain

Adult neural stem cells (NSCs) are not ubiquitously distributed throughout the adult mammalian brain but rather restricted to specific areas with a unique and diverse microenvironment that supports neurogenesis, the neurogenic niches [32]. During mammalian brain development, multipotent NSCs expand through symmetric self-renewing divisions to produce two identical daughter cells [33, 34]. Later, they divide asymmetrically to produce NSCs to maintain the stem cell pool, and an intermediate progenitor cell (IPC) that commits to differentiation [35]. Of note, once NSCs have divided and differentiated into various types of neural cells, they will enter apoptosis, terminal division, or senescence, meaning they will either die or stop dividing, resulting in a reduced number of NSCs. This explains why in the adult brain there are only a few NSCs remaining [36]. IPCs undergo a series of cell divisions, promptly expanding and differentiating into neuroblasts or glial cells, depending on the specific signals they receive from their microenvironment [37]. Neuroblasts, which are immature neurons, are lineage-restricted [38]. These cells move from their niche to their intended destination in the brain (which, to some extent, depends on the niche from which they migrate [39]) where they differentiate into mature neurons by establishing connections with other neurons through the formation of synapses [40]. The functional specialization of the new neurons is dependent on the function of the region in which they were integrated [41]. Besides NSCs and IPCs, other cell types coexist in neurogenic niches, such as neuroglia (astrocytes and oligodendrocytes), immune cells (microglia and macrophages), and endothelial cells [38, 42]. The phenotypical richness of these niches creates a special and unique environment that is optimized for the regulation of NSC self-renewal and differentiation. This tight control is assured by the extracellular matrix, short and long-range signalling factors, and direct signaling across gap junctions [43, 44].

**Fig. 1:**
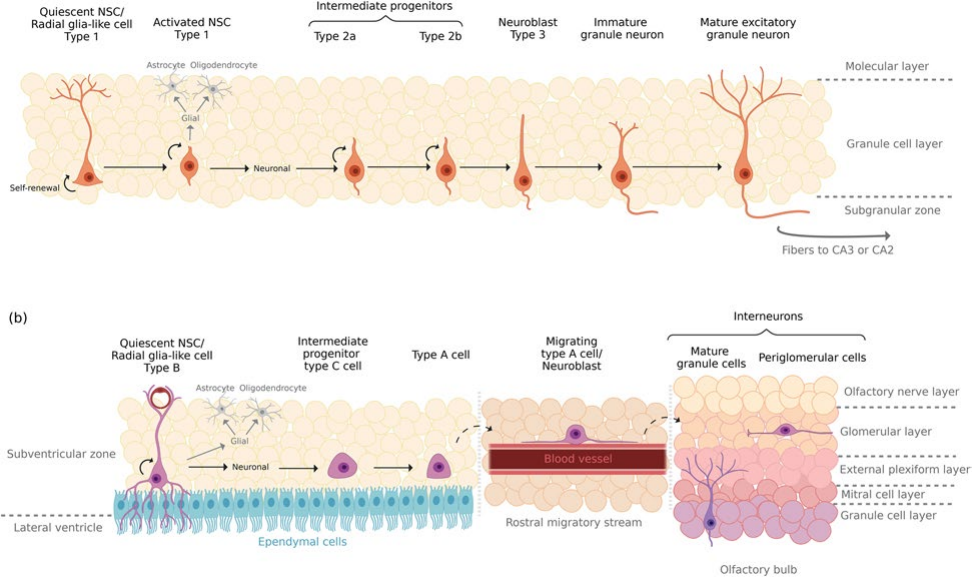
Schematic representation of the different stages of adult neurogenesis in the main neurogenic niches. **a** In the SGZ, once activated, type-1 cells exit quiescence and may self-renew by symmetric cell division, or undergo asymmetrical division to generate type-2 cells [49]. Of note, these cells can commit to a glial lineage, which can give rise to either astrocytes or oligodendrocytes. But, if committed to a neuronal lineage, type 1 cells can differentiate into two distinct subsets of cells: type-2a and type-2b [50]. The latter are more differentiated and fate-specific and, therefore, they result in type-3 cells, which are neuroblasts with little proliferative activity that experience dendritic and axonal elongation [47]. Neuroblasts further differentiate into immature dentate granule cells. These migrate through the granule cell layer and extend their axonal projections towards the CA3 or CA2 regions of the dentate gyrus, which allows their maturation into excitatory granule neurons [47]. **b** In the SVZ, an astrocyte-like population of NSCs, named type B cells, are capable of producing neurons, astrocytes, and oligodendrocytes [51]. Upon activation, these cells start to divide asymmetrically for self-renewal or to generate rapidly dividing transit amplifying progenitors. These intermediate progenitor cells are known as type C cells, and they divide three times before differentiating into neuroblasts (type A cells) [52]. Migrating A cells form elongated cell aggregates (migratory chains, not shown) that migrate towards the olfactory bulb along the rostral migratory stream [53, 54]. After arriving at the olfactory bulb, these clusters of neuroblasts dissociate into individual cells and migrate into different structures within the olfactory bulb where they mature [55]. In the granule cell layer, interneurons mature into granule cells, whereas in the glomerular cell layer, they mature into periglomerular cells [48]. Created with Biorender.com

The hippocampal SGZ and the SVZ are the two most studied and well-described neurogenic niches in the adult mammalian brain, sometimes being referred to as the “classical” or “traditional” neurogenic zones [7, 45]. In both areas, the neurogenic process has been extensively characterized [46]. Under physiological conditions, neurogenesis in the SGZ generates only one type of neuron, the granule cells, which represent the main glutamatergic excitatory neurons of the DG [47]. In the SVZ, neurogenesis results in granule cells and periglomerular cells, upon migration to the OB [48] (Fig. 1).

Compared to the hippocampus, the functional relevance of SVZ neurogenesis is poorly understood. Therefore, this topic should be addressed in near future studies, especially the relationship between olfactory and social behaviour and memory.

Even though most of the research on adult neurogenesis has been focused in the SGZ and SVZ, there is limited evidence of other neurogenic niches distributed throughout the adult mammalian brain. To date, most of these proposed neurogenic niches have only been identified in animal studies. For example, neurogenesis in the hypothalamic niche is thought to be functionally related to energy balance and other hypothalamic homeostasis mechanisms, namely fat storage and metabolism [56, 57], as well as sexual and social behaviour [58]. It has been hypothesized that neurogenesis in the amygdala functions as a mechanism for stress regulation and fear condition [59, 60].

Neurogenesis in the *substantia nigra* is still not fully established, nonetheless, it has been found to be active in animal models of Parkinson’s disease (PD) [18], and in post-mortem brains of humans with the same condition [61] where the additional generation of new neurons may serve as a compensatory mechanism. Additionally, Lie et al. found a population of NG2+ glial progenitor cells in the basal *substantia nigra* of rodents [62]. These cells have the potential to differentiate into neurons under specific conditions, suggesting the possibility of neurogenesis in that specific brain region. Furthermore, their presence in rodents hints at the potential for neurogenesis in the human *substantia nigra* as well.

Likewise, adult neurogenesis in the striatum is limited under normal physiological conditions but is increased in response to pathological stimuli, such as stroke, ischemia and injury [45]. Little is known about the functional relevance of neurogenesis in the cortex, cerebellum and habenula. However, it is suggested that neurogenesis in the habenula is responsible for multisensory processing such as visual, olfactory, mechanosensory, and aversive stimuli [63].

The source of the adult-born neurons in these novel neurogenetic niches is still a matter of debate. Some studies report that the new neurons in these niches originated from precursor cells that migrate from distant pools of NSCs, namely the SVZ [64]. While other authors state that the neurogenic niches circuitry is populated by endogenous dividing IPCs [65]. Nonetheless, the emergence of these novel niches offers an exciting possibility for better understanding the functional implications of neurogenesis on the adult mammalian brain in health and disease.

## Strategies to Promote Neurogenesis

Impaired adult neurogenesis is a common hallmark in many neurodegenerative diseases, which are a group of nervous system disorders characterized by the progressive loss of neuronal structures and functions, in a process termed neurodegeneration [66]. These include AD, PD, Huntington’s disease (HD), and Lewy bodies dementia [67], among others.

Dysregulated neurogenesis can contribute to the development of these disorders through two main ways: reduction in new-born neurons production, or due to their abnormal maturation and subsequent impaired integration [68]. However, given the capacity of neurogenesis to make up for neuron loss and fix damaged neural circuits, researchers aim to promote or at least preserve, the limited regenerative potential of the adult brain, to potentially treat the neuronal and cognitive loss observed in neurodegenerative diseases [69].

Research has uncovered key factors that boost innate neurogenesis, such as neurotrophic factors [70], neurotransmitters [71], transcriptional programs [72], inflammatory cytokines [73], and hormones [74]. These factors may be modulated through lifestyle changes [75], neurostimulation [76], pharmacological stimuli [77], or hormone therapy [78]. These strategies aim to create an environment that is conducive to neurogenesis and encourage the brain to produce new neurons on its own.

However, at the late stages of neurodegenerative diseases, there is an exacerbated neuronal loss and limited rescuing potential. In these cases, the damage is too severe to be repaired simply by stimulating the brain’s endogenous regenerative mechanisms [79]. Thus, researchers are seeking other strategies to re-establish neurogenesis [69] resorting to exogenous approaches such as cell-based therapies [80]. Therefore, in the following sections, these two different approaches will be explored as a mean to treat or at least attenuate the symptoms of different neurodegenerative diseases.

### Endogenous Neurogenesis Stimulation

Different ways to expand the brain’s physiological neurogenic potential are being studied. Indeed, many pro-neurogenic approaches have already proved to increase basal levels of neurogenesis in adult animals with some correlated improved cognition [81]. However, more research is needed to optimize existing therapies and find novel ones. Therefore, in this section, different strategies to stimulate endogenous neurogenesis will be discussed in detail.

#### Electric and Magnetic Neurostimulation

During the process of brain development and to some extent in adulthood, immature neurons migrate from their origin to their site of maturation, partly due to bioelectricity [76, 82]. Because it is still in its infancy, there is limited evidence of electric, magnetic, or electromagnetic fields being able to promote neurogenesis merely by inducing electrical currents [76]. However, different studies have presented promising results [83]. Additionally, this type of therapy has the advantage of being non-systemic, unlike other treatment options [84].

**Fig. 2:**
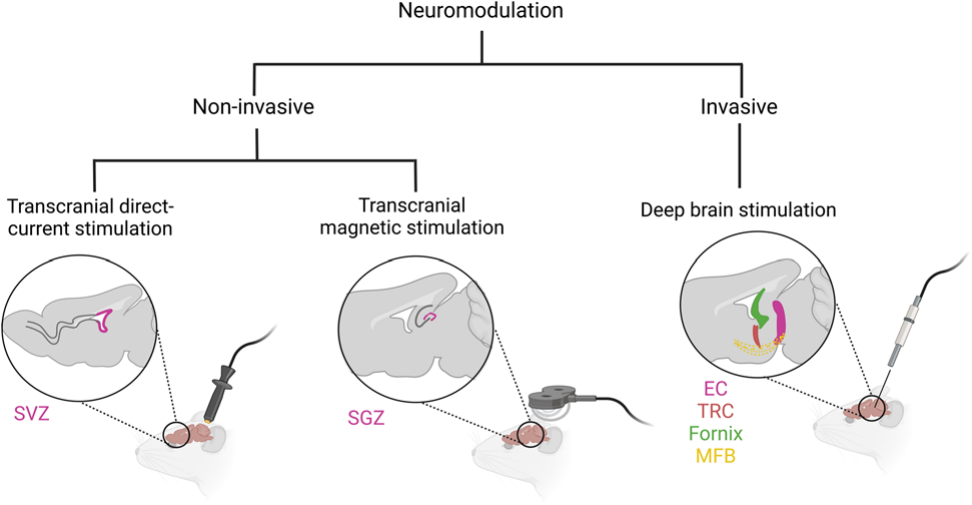
Examples of non-invasive and invasive neurostimulation techniques that stimulate neurogenesis through the modulation of a variety of brain structures. Non-invasive techniques such as transcranial direct-current stimulation (tDCS) and transcranial magnetic stimulation (TMS) have a direct influence on conventional neurogenic niches, such as the subventricular zone (SVZ) of the lateral ventricles, and the subgranular zone (SGZ) of the hippocampus, respectively. Deep brain stimulation, which is an invasive neuromodulation therapy, indirectly stimulates neurogenesis in the SGZ by influencing adjacent structures near the hippocampus. EC, entorhinal cortex; MFB, medial forebrain bundle; TRC, thalamic reticular nucleus. Created with Biorender.com

Currently, there are different techniques to provide brain electrical and magnetic stimulation, some of which have been explored for their potential to promote neurogenesis [85]. Those that have provided the most promising pro-neurogenic results include noninvasive techniques such as transcranial direct current stimulation (tDCS), transcranial magnetic stimulation (TMS), and invasive deep brain stimulation (DBS) [86] (Fig. 2).

The underlying mechanisms by which these methods promote neurogenesis remain poorly understood. However, recent advances in the field have been reviewed in [87], providing valuable insight into the possible molecular mechanisms behind neurostimulation in vitro.

TDCS involves the application of low-level electrical currents (typically less than 2 mA) to the scalp [88], which then penetrate the skull to stimulate the underlying brain tissue, promoting neurogenesis in both healthy and pathologic conditions [89]. Different studies with mice and cats reported that tDCS promoted the proliferation of NSCs [90], their differentiation into neuroblasts [91], and enhanced their mobility [92]. Additionally, tDCS prevented Amyloid-β 1-42 (Aβ 1-42) aggregation in AD patients [93]. However, this pro-neurogenic effect only appears to be unanimous in the SVZ, but not SGZ [89, 94].

TMS is a newer form of neurostimulation that induces depolarization or hyperpolarization of neurons through magnetic pulses [95, 96]. Different TMS methods have been studied for their neuromodulatory potential, but low- and high-frequency repetitive TMS has presented the most positive effects on neurogenesis [95, 97], especially in the hippocampus [98]. Immunohistochemistry assays confirmed that repetitive TMS with frequencies in the range of 0.5–25 Hz appears to induce neurogenesis by upregulating the expression of brain-derived neurotrophic factor (BDNF), which is a neurotrophin that promotes NSCs migration and proliferation via its receptor, tropomyosin-related kinase B (TrkB) [99, 100]. Additionally, it also upregulates nerve growth factor (NGF) [101], neurotransmitter GABA [102], synaptic proteins such as synaptophysin [99], and receptors for N-methyl-D-aspartic (NMDA) [101, 103] and α-Amino-3-hydroxy-5-methyl-4-isoxazolepropionic acid (AMPA) [103]. Finally, it enhances the expression of growth factors with vasculotrophic activity, such as vascular endothelial growth factor (VEGF) and transforming growth factor (TGFβ) [103], promoting the proliferation of blood vessels which support the newly generated neurons with blood supply [104]. Although it has been found to increase neurogenesis in animal models of neurodegenerative diseases, its effect in healthy animals is still not well understood, as one study suggests that TMS raises neurogenesis [105], while another reports no effects [106]. Therefore, more studies are needed to clarify this issue.

DBS is an invasive neuromodulatory therapy that consists of implanting electrodes in specific regions of the brain and delivering alternating currents to modulate the brain’s activity [107]. Although this technique has been successfully implemented in patients with PD and other movement disorders for many years, recently, an unexpected improvement in memory was observed during a DBS procedure for obesity treatment [108], which expanded the potential use of DBS to treat disorders affecting the hippocampus [109]. In rodent models, acute stimulation of structures with anatomical connections with the hippocampus has successfully promoted circuit function and neurogenesis, which were correlated with improved exploratory behavior and hippocampal memory [109, 110]. These include structures such as the entorhinal cortex [107, 111], thalamic anterior nucleus [112, 113], thalamic reticular nucleus [114], medial forebrain bundle [115], and fornix [109] (Fig. 2).

Although the above data provide evidence that electric and magnetic stimulation are efficient strategies to treat patients suffering from cognition dysfunctions and that the effects of neurostimulation on neurodegeneration symptoms may involve increasing neurogenesis [116], this is still an active area of research with some mixed results [117, 118]. Therefore, optimal level of stimulation is still being explored [119].

#### Lifestyle Changes

Following a healthy lifestyle, such as practicing physical exercise, dietary restriction, and being exposed to environmental stimuli are effective and non-evasive strategies that induce innate neurogenesis by promoting the expression of endogenous neurotrophic factors [81, 120].

**Fig. 3:**
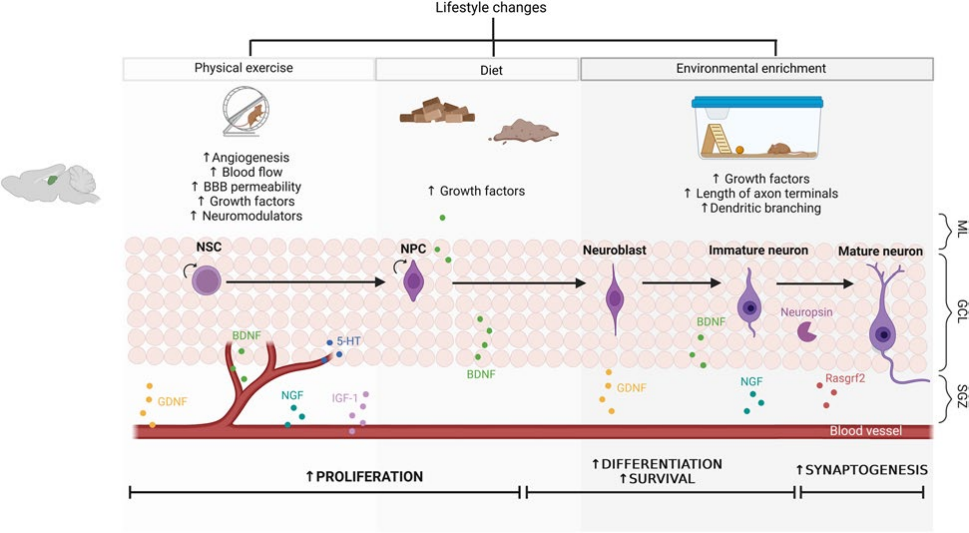
Lifestyle changes that promote hippocampal neurogenesis. These changes, which include intense physical exercise, dietary restriction, and environmental enrichment, have shown a pro-neurogenic impact in different niches, however, a more pronounced effect can be observed in the SGZ. Each activity seems to act on distinct phases of the neurogenic process. Physical exercise, specifically during the proliferation phase, correlates with angiogenesis and increased permeability of the blood-brain barrier. This leads to the release of growth factors and neuromodulators into the SGZ niche, ultimately stimulating the proliferation of NSCs. Dietary changes, such as calorie restriction, stimulate the release of growth factors, particularly BDNF, which plays a crucial role in the progression of the neurogenic process. Environmental enrichment primarily impacts the later phases of neurogenesis. It promotes the survival of newborn neurons and induces morphological changes associated with the maturation of these new cells. BDNF, brain-derived neurotrophic factor; GCL, granule cell layer; GDNF, glial-cell line-derived neurotrophic factor; IGF-1, insulin-like growth factor 1; ML, molecular layer; NGF, nerve growth factor; NPC, neural progenitor cell; NSC, neural stem cell; Rasgrf2, Ras Protein Specific Guanine Nucleotide Releasing Factor 2; SGZ, subgranular zone; ↑, increase. Created with Biorender.com

By studying humans, mice, and rats, it has become clear that in the SGZ, intense exercising (such as swimming and running) leads to an increase in angiogenesis [121], cerebral blood flow [122], blood-brain barrier permeability [123], and the expression of neurotrophins and growth factors [124]. Those include BDNF [125], NGF [126], glial-cell line-derived neurotrophic factor (GDNF) [127], VEGF [128], and insulin-like growth factor 1 (IGF-1) [129], all of which have shown to influence hippocampal neurogenesis [124]. Additionally, physical exercise was also reported to increase hippocampal extracellular levels of the neuromodulator serotonin (5-HT) [130]. Due to changes in vasculature, these molecules are more readily delivered to the hippocampus which ultimately promotes the proliferation of new neurons in the DG [81] (Fig. 3).

These results have been associated with improved spatial memory, neuronal plasticity, and learning, which appears to indicate that consistent physical activity may improve cognitive function through the promotion of neurogenesis [131].

While physical exercise improves SGZ local environment, it appears to serve a smaller role in the SVZ [132]. Indeed, there is less evidence of SVZ neurogenesis responding to exercise [133] which is accompanied by some conflicting reports [134, 135]. Surprisingly, a recent study showed that voluntary running-induced neurogenesis in the hypothalamus and ependymal lining of the third ventricle of rats, correlated with the increase of fibroblast growth factor-2 (FGF-2) [136].

Environmental enrichment (EE) is another lifestyle factor with strong connections to hippocampal neurogenesis. It refers to an experimental setting in which animals are confined in an environment that provides sensory, social, and motor stimulation for optimal physiological and psychological well-being [137, 138]. Several studies have proved the neurogenic benefits of EE in rodents [139], which also translate into animal models of neurodegenerative diseases [140]. However, it is crucial to point out that the effects of EE are age-dependent since its neuroprotective strategy depends on its exposure from an early age [141]. Just as physical exercise, EE also increases BDNF [142], NGF [143], GDNF [144], and VEGF [145] which are the most well-established pro-neurogenic factors [146]. But other recently discovered mediators include neuropsin (Klk8) [147] and Ras Protein Specific Guanine Nucleotide Releasing Factor 2 (Rasgrf2) [148]. Finally, this pro-neurogenic strategy has also proved to induce morphological changes in neurons — it lengthens axon terminals [149], enhances dendritic branching [150], and increases synaptogenesis [151] (Fig. 3). However, the exact mechanisms causing these effects remain unknown [152].

In sum, both exercise and EE predominantly promote innate hippocampal neurogenesis; however, physical activity is considered to mainly enhance cell proliferation, while EE promotes new cell survival [153], which overall delays the progression of neurodegenerative diseases [141].

Another environmental factor that can also modulate adult neurogenesis is diet. Neurogenesis is influenced by different aspects, such as caloric intake, meal texture, and content [154]. Briefly, reduced calorie intake has been shown to enhance neuronal proliferation and differentiation in the DG of rodents [155], which correlates with increased BDNF levels [156]. In fact, in experimental animal models of AD, PD, and HD, calorie restriction improved the resistance of neurons to dysfunction and death [157]. But beyond diet, mastication also seems to influence neurogenesis [158]. Indeed, loss of teeth was identified as a risk factor for the development of senile dementia and AD [159]. This is in agreement with different studies that compared rodents fed with a soft and hard diet and found that rodents that chew food have increased progenitor cell proliferation in the DG [160], the hypothalamus [161], and higher neuroblast migration to the OB [162]. Also, in the DG, chewing correlated with increased levels of BDNF [160], and decreased plasma corticosterone levels [163], which is a glucocorticoid that supress the proliferation of neural precursors in the DG [164].

Still regarding diet, several nutrients have been recognized as potential neuromodulators [165]. For example, diets rich in refined sugars and fat have been shown to significantly impair neurogenesis in rodents [166]. Specifically, a high dietary intake of saturated fats has been reported to impair hippocampal neurogenesis by increasing corticosterone levels [154, 167]. Conversely, the consumption of plant and animal foods rich in specific dietary compounds can enhance neurogenesis [165]. These include folate (vitamin B9) [168], cobalamin (vitamin B12) [169], fat-soluble vitamins (e.g., vitamin E [170] and vitamin A [171]), polyunsaturated fatty acids (e.g., omega-3) [172], polyphenols (e.g., curcumin [173] and resveratrol [174]), and minerals (e.g., zinc) [175]. Thus, practicing a healthy and diverse diet may be an effective strategy to promote neurogenesis and consequently improve neurodegeneration in aged individuals or patients suffering from neurodegenerative diseases. However, the effects of dietary compounds on neurogenesis appear to be greatly influenced by their dose [176, 177]. As dietary intake presents challenges in controlling dose, pharmacological supplementation appears to be a more viable approach. Subsequently, the following section will delve into the impact of supplementing these compounds in greater detail.

#### Pharmacological Manipulation

The pharmacological intervention for impaired neurogenesis in neurodegenerative diseases aims to create a compensatory mechanism for neuronal loss. Currently, there is not a single neurogenic drug for the treatment of neurodegenerative diseases. Nonetheless, researchers are focused on finding safe and effective biochemical and pharmacologic agents that can restore or even increase neurogenesis in patients suffering from neurodegeneration. Table 1 lists the neurogenic stimulatory actions of different pharmacological agents on different animal models of neurodegenerative diseases.

**Table 1:** The influence of different pharmacological agents on neurogenesis of animal models with neurodegenerative conditions

Molecule	Dose, duration, and administration mode	Disease model	Brain tissue	Results					References
				Molecular and cellular level			Functional recovery	Molecular mechanism	
1. Bioactive compounds									
Resveratrol	50 mg/kg/day from gestation day 1 to post-natal day 21, by gavage	SD rats with neurodevelopment issues induced by lead exposure	Hippocampus	↑ NSC proliferation and ↓ apoptosis of new neurons (↑ marker of NSC proliferation Ki67, and marker for mature neurons NeuN)			↑ Spatial learning and memory capabilities	Activation of SIRT1/CREB/BDNF signaling pathway	[178]
Curcumin	150 mg/kg/day for 42 days, intragastrically	C57BL/6 transgenic APP/PS1 mice model of AD	Hippocampus	↑ NSC proliferation (↑ cell cycle progression proteins CDK4 and cyclin D1, and marker of NSC proliferation BrdU)			↑ Spatial learning and memory capabilities	Activation of Notch signaling pathway	[179]
	25, 50, and 100 mg/kg/day for 30 days, by gavage	Wistar rats’ model of a dementia of AD type, induced with streptozotocin	SGZ	Did not restore neurogenesis (no effect on markers Ki67 and marker of immature neurons DCX)			Prevented impairments in recognition memory but not spatial memory	NA	[180]
	5, 10, and 20 mg/kg/day, from post-natal day 28 to 49, I.P.	Wistar rats’ model of AD, induced with injections of Aβ (1-42)	SGZ and SVZ	↑ NSC proliferation and differentiation (↑ genes involved in cell proliferation and neuronal differentiation: Reelin, Nestin, Pax6, neurogenin, neuroD1, neuregulin, neuroligin, and Stat3)			↑ Learning and memory capabilities	Activation of Wnt/β-catenin signaling pathway	[181]
Retinoic Acid	20 mg/kg, 3 times a week, for 4 weeks, I.P.	APPswe/PS1_M146V_/tau_P301L_ triple transgenic mice model of AD	SGZ	↓ Neuroinflammation (↓ microglia activation) ↑ NSC proliferation (↑ marker of NSC proliferation PCNA)			NA	NA	[182]
	Loaded in nanoparticles, 100 ng/ml, single intrastriatal injection	C57BL/6 mouse model of PD, induced with MPTP	*Substantia nigra* and striatum	↑ Development and survival of dopaminergic neurons in the *substantia nigr*a (↑ TH staining, and ↑ mRNA and protein expression of Nurr1 and Pitx3) ↑ Dopaminergic fiber striatal innervations in the striatum (↑ TH+ immunoreactive fibers)			NA	NA	[183]
Ginsenoside (Rg1)	20 mg/kg/day for 28 days, I.P.	Brain aged SD rat through D-galactose	Hippocampus	Protection of the hippocampus against senescence and delay of NSCs’ telomere shortening (↓ biomarkers for aging cells: SA-β-gal and telomerase) ↓ Astrocyte activation and ↓ neuroinflammation (↓ proinflammatory cytokines IL-1β, IL-6, and TNF-α) ↓ Oxidation (↑ antioxidant enzymes SOD and GSH-Px) ↑ NSC differentiation into neurons (↑ markers of NSCs and NPCs Sox2, and Nestin, and differentiation marker β-tubulin III)			↑ Spatial learning and memory capabilities	NA	[184]
	20 mg/kg/day for 28 days, I.P.	Brain aged C57BL/6 mice through D-galactose	SGZ	↑ NSC proliferation (↑ Sox2 and Nestin) ↓ Oxidation (↓ markers of oxidative stress MDA and ROS, but ↑ of antioxidant enzymes SOD and GSH-Px)			↑ Spatial learning and memory capabilities	Inhibition of the Akt/mTOR signaling pathway	[185]
Ginsenoside (Rb1)	10 mg/kg/day for 30 days, I.P.	SD rats’ model of AD, induced with injections of Aβ (1-40)	All brain tissue	↑ NSC proliferation and differentiation (↑ markers of NSCs and NPCs, Nestin and GFAP, and marker of immature neurons NSE)			NA	NA	[186]
Gintonin	50 or 100 mg/kg/day for 21 days, orally	C57BL/6 double transgenic AβPP_swe_/PSEN1_dE9_ mice model of AD	Hippocampus	↑ NSC proliferation and differentiation into both neurons and astrocytes (↑ LPA receptors, and markers BrdU and NeuN)			NA	NA	[187]
	50 or 100 mg/kg/day for 4 weeks, orally	Brain aged C57BL/6 mice through D-galactose	Hippocampus	↑ NSC proliferation, differentiation, and neuronal maturation (↑ Ki67, BrdU, DCX and NeuN) ↑ Long-term potentiation ↑ Activation of CREB ↑ LPA1 receptor ↓ Oxidation (↓ ROS)			↑ Object location memory	NA	[188]
Oleanolic acid	10 mg/kg/day for 28 days, I.P.	APP/PS1 double transgenic mice model of AD	Hippocampus	↑ NSC proliferation (↑ Sox2, BrdU, and NeuN) ↑ Number of healthy neurons			↑ Spatial learning and orientation ↓ Anxious behavior	Activation of Wnt/GSK-3β/β-catenin signaling pathway	[189]
Protopanaxadiol	10 mg/kg/day for 28 days, I.P.	APP/PS1 double transgenic mice model of AD	Hippocampus	↑ NSC proliferation (↑ Sox2, BrdU, and NeuN) ↑ Number of healthy neurons			↑ Spatial learning and orientation ↓ Anxious behavior	Activation of Wnt/GSK-3β/β-catenin signaling pathway	[189]
Baicalin	10 mg/kg/day for 30 days, I.P.	SD rats’ model of AD, induced with injections of Aβ (1-40)	All brain tissue	↑ NSC proliferation (↑ Nestin)			NA	NA	[186]
Rosmarinic acid	16 mg/kg/day for 15 days, orally	BALB/c mice model of AD, induced with injections of Aβ (1-42)	Hippocampus	Preservation of neurons morphology and density ↑ Cells’ proliferation, migration, maturation (↑ DCX, NeuN, and synaptic marker synaptophysin)			↑ Spatial learning and memory capabilities ↓ Depressive and anxious behavior	NA	[190]
Ursolic acid	40 mg/kg/day for 15 days, orally	BALB/c mice model of AD, induced with injections of Aβ (1-42)	Hippocampus	Preservation of neurons morphology and density ↑ Cells’ proliferation, migration, maturation (↑ Ki67, DCX, NeuN, and synaptic marker synapsin I)			↑ Spatial and object recognition memory ↓ Depressive and anxious behavior	NA	[190]
Egb 761	*Ad libitum* daily intake of 100 mg/kg	C57BL/6J double transgenic APP_swe_/PS1_ΔE9_ mice model of AD	Hippocampus	↑ NSC proliferation and maturation of new-born neurons (↑ BrdU, NeuN, and immature neuronal marker NCAM-180, but not GFAP) ↑ pCREB ↓ Aβ aggregates			NA	NA	[191]
Butylphthalide	15 mg/kg, 5 days/week, for 12 weeks, by gavage	B6C3 double transgenic APP_swe_/PSEN1_dE9_ mice model of AD	Hippocampus	↑ NSC proliferation (↑ marker BrdU and neurotrophins BDNF, and NGF) ↑ Activation of TrkA and TrkB receptors, CREB and Akt			↑ Spatial learning	Activation of BDNF/TrkB/CREB/Akt signaling pathway	[192]
2. Antidepressants									
Fluoxetine	20 mg/kg/day for 10 weeks, I.P.	R6/1 transgenic HD mice	Hippocampus and SVZ	↑ NSC differentiation (↑ BrdU and NeuN) ↑ NSC survival, but no effect on the net increase of new cells ↑ Volume of the DG			↑ Spatial memory ↓ Depressive behavior No effect on motor activity	NA	[193]
	10 mg/kg/day for 15 days, orally	C57BL/6 mice model of PD, induced with injections of rotenone	Hippocampus, prefrontal cortex, and *substantia nigra*	↓ Neuroinflammation in the hippocampus and prefrontal cortex (↓ proinflammatory cytokines IL-1β, phospho-NF-kB) ↓ Hippocampal cell death (↓ oxidation marker iNOS, and ↓ cell death related proteins PARP-1 and caspase-3) ↑ NSC proliferation and synaptic plasticity in the hippocampus (↑ Ki67, and markers of synaptic plasticity pCREB and BDNF, but no effect on NeuN) No effect on dopaminergic neurons No effect on TH staining in the s*ubstantia nigra*			↓ Depressive behavior ↓ Motor deficits	NA	[194]
	18 mg/kg/day for 11 months, orally	PS1_M146V_/APP_swe_/tau_P301L_ triple transgenic mice model of AD	Hippocampus	No effect on neurogenesis (no effect on BrdU, NeuN, or BDNF)			Motor impairment and no effect on neuronal differentiation	NA	[195]
	10 mg/kg/day for 5 weeks, I.P.	APP_swe_/PSEN1_dE9_ double transgenic mice model of AD	Hippocampus	↑ Neurons in the DG, but no effect in the CA1 and CA3 regions ↓ Aβ aggregates Inhibition of GSK-3β and stabilization of β-catenin			↑ Spatial learning	Activation of Wnt signaling pathway	[196]
Amitriptyline	1 mg/kg/day for 4 months, orally	PS1_M146V_/APP_swe_/tau_P301L_ triple transgenic mice model of AD	Hippocampus and cortex	↑ NSC differentiation (↑ BrdU, and NeuN in the DG) ↑ NSC maturation (↑ synaptic markers synaptophysin, synapsin I, PSD95, and spinophilin on the hippocampus, but no effects on the cortex) Direct activation of TrkB receptor ↑ Aβ aggregates (insoluble form) in the hippocampus, subiculum, CA1, and amygdala and tau deposition (insoluble form) in the hippocampus			↑ Spatial learning and memory	NA	[197]
	10 mg/kg/day for 38 days, I.P.	Wistar rats’ model of LBD, injected with the SNCA gene and Aβ (1-42)	Hippocampus and *substantia nigra*	↑ NSC proliferation in the DG (↑ BrdU) ↑ Neuronal densities in the CA1 region and *substantia nigra (*↑ TH and Nissl staining) ↓ Accumulation of α-synuclein in the DG			↑ Object recognition	NA	[198]
	16 mg/kg/day for 6 weeks, orally	B6C3 transgenic N171-82Q mice model of HD	Striatum, cortex and whole brain	↑ NSC differentiation and maturation in all tissues In the striatum: ↑ Neurotrophin BDNF via CREB ↑ DCX and NeuN ↑ Presynaptic markers (synaptophysin) but no effect on postsynaptic markers (PSD95) In both tissues: ↓ mHTT protein in the striatum and cortex Whole brain: ↑ Expression of factors related to neuronal development (Sparc, Nyap) ↓ Neurodegenerative cross-linking (Tgm2)			↑ Motor function	Activation of the BNDF-TrkB signaling pathway	[199]
Lithium	10 mg/kg/day for 5 weeks, I.P.	Transgenic CRND8 mice model of AD	Hippocampus and cortex	↑ NSC proliferation and differentiation in the SGZ (↑ BrdU, DCX, and NeuN) ↑ Survival of new-born neurons and migration into the DG ↓ Glia activation and neuroinflammation (↓ IBA-1 and GFAP) ↓ Aβ and tau aggregates in both tissues Inhibition of GSK-3β			↑ Spatial learning and memory in 3- but not 7-month old Tg mice	Activation of Wnt pathway	[200]
3. Small molecules									
P7C3-S243	10 mg/kg/day for 9 and 18 months, I.P.	Transgenic F344 rats’ model of AD	Hippocampus and cortex	↑ NSC proliferation and differentiation (↑ BrdU and NeuN) No effect on glia activation ↓ Neurodegeneration (↑ number of neurons) No effect on Aβ and tau deposition, or neuroinflammation			↓ Depressive behavior ↑ Relearning abilities	NA	[201]
NNI-362	3 mg/kg/day for 4 weeks, I.P.	Ts65Dn mice model of early-stage AD	Hippocampus	↑ NSC proliferation and survival (↑ Ki67 and BrdU) ↑ Phosphorylation of p70S6 kinase			↑ Object recognition	Suggested activation of mTOR signaling pathway	[202]
4. Glutamate receptor antagonists									
Dizocilpine (NMDAr antagonist)	0.2 mg/kg/day for 28 days, I.P.	SD rats’ model of PD, induced with injections of 6-OHDA	Hippocampus, striatum, and *substantia nigra*	↑ Hippocampal NSC self-renewal capacity, proliferation, long-term survival, and differentiation (↑ Sox2, Ki67, BrdU, Nestin, and NeuN) ↑ Dendritic arborization of hippocampal immature neurons ↑ Neuronal migration in the hippocampus ↑ Dopaminergic neurons in the striatum and *substantia nigra* (↑ transcription factors involved in the development of dopaminergic neurons Nurr-1 and Pitx-3) No effect on the proliferation of oligodendrocyte progenitor cells in the hippocampus (no alterations of marker Olig-2) ↑ Myelination (↑ MBP expression)			↓ Depressive and anxious behavior ↑ Motor function	Activation of Wnt/β-catenin signaling pathway	[203]
Memantine (NMDAr antagonist)	20 mg/kg/day for 4 months, orally	Tg4-42^hom^ mice model of AD	Hippocampus	↑ New-born neurons (↑ DCX) ↑ Pyramidal neurons in the CA1 region			↑ Object recognition and spatial memory No effect on anxiety levels ↓ Motor deficits	NA	[204]
BCI-632 (Group II mGlur antagonist)	Daily oral administration for 3 months Dose NA	Dutch APP transgenic E693Q mice model of AD	Hippocampus and cortex	↑ Surviving, new-born neurons in the hippocampus (↑ BrdU and NeuN) ↓ Aβ monomer and oligomer levels			↓ Anxious behavior ↑ Working memory, spatial learning, and social recognition	NA	[205]
5. Other drugs									
Metformin	200 mg/kg/day for 15 days, orally	C57BL/6 mice model of PD, induced with injections of rotenone	Hippocampus, prefrontal cortex, and *substantia nigra*	↓ Glial cells and neuroinflammation (proinflammatory cytokines IL-1β, phospho-NF-kB) in the hippocampus and prefrontal cortex No significant reduction on hippocampal cell death (no effect on PARP-1 and caspase-3) ↑ NSC proliferation but no effect on the number of mature neurons (↑ Ki67, but no effect on NeuN) in the hippocampus No effect on synaptic plasticity No (unaltered pCREB and BDNF levels) in the hippocampus ↑ Dopaminergic neurons (↑ TH staining) in the *substantia nigra*			↓ Depressive behavior ↓ Motor deficits	NA	[194]
	500 mg/kg/day for 21 days, orally	Swiss-albino mice model of PD, induced with injections of MPTP and probenecid	Midbrain, *substantia nigra*	Protection of dopaminergic neurons in the *substantia nigra* through antioxidant defense (↑ SOD, CAT, and GSH) and reduced oxidation (↓ LPO) in the midbrain ↑ Dopaminergic neurons (↑ TH staining and BDNF levels) in the *substantia nigra*			↓ Motor deficits	NA	[206]
	200 mg/kg/day for 14 days, I.P.	B6C3 double transgenic APP_swe_/PS1_dE9_ mice model of AD	Hippocampus	↑ Number of mature neurons (↑ NeuN) in the CA1 region ↓ Aβ plaques ↓ Neuroinflammation (↑ anti-inflammatory IL-4, ↓ pro-inflammatory IL-1β, TNF-α) ↑ p-AMPK and ↓ p-mTOR, p-26K, p-P65 NF-kB, and Bace-1 in microglia and astrocytes ↓ Neuronal apoptosis ↑ NSC proliferation, differentiation, and migration (↑ BrdU and DCX)			↑ Spatial memory	Activation of AMPK/mTOR/S6K/Bace1 and AMK/P65 NF-kB signaling pathways	[207]
Sovateltide	0.005 mg/kg three times at 2-h intervals on days 1, 3, and 6 every month until endpoints (3-, 6-, and 12-months age), intravenous	APP/PS1 double transgenic mice model of AD	Hippocampus	↑ Neurogenesis (↑ NeuroD1, DCX, NeuN and synaptic proteins synapsin I, synaptophysin, and PSD95) ↓ Aβ plaques ↑ Mitochondrial fusion proteins (Mfn1, Mfn2, Opa1) but ↓ in fission proteins (Drp1, Fis1)			↑ Learning and memory	Activation of ET_B_ receptor signaling pathway	[208]
Panobinostat (LBH589)	0.001, 0.01, 0.1 and 1 mg/kg, every 48 h, from post-natal day 8 to 20, I.P.	Double transgenic mice model of HD	Striatum	↑ Neurogenesis (↑ DCX and ↑ rostral migratory stream volume) ↑ Dopamine pathway related-gene (RasGRP2) ↑ Striatal development markers (DARPP-32 and PPP1R7)			↓ Anxious behavior	NA	[209]

Abbreviations: *AD*, Alzheimer’s Disease; *Akt*, protein kinase B; *AMPK*, AMP-activated protein kinase; *Aβ*, Amyloid-β; *APP*, amyloid precursor protein; *Bace 1*, Beta-secretase 1; *BDNF*, brain-derived neurotrophic factor; *BrdU*, bromodeoxyuridine; *CA1*, *cornu ammonis* 1; *CA3*, *cornu ammonis* 3; *CAT*, catalase; *CDK4*, cyclin-dependent kinase 4; *CREB*, cyclin-dependent kinase 4; *CREB*, cAMP response element-binding protein; *DCX*, doublecortin; *DG*, dentate gyrus; *Drp1*, dynamic-related protein 1; *ET*_*B*_, endothelin receptor type B; *Fis 1*, mitochondrial fission 1 protein; *GFAP*, glial fibrillary acidic protein; *GSH*, glutathione; *GSH-Px*, glutathione peroxidase; *Gsk-3β*, glycogen synthase kinase-3β; *HD*, Huntington’s Disease; *IBA-1*, ionized calcium-binding adaptor molecule 1; *IL-1β*, interleukin 1β; *IL-4*, interleukin 4; *IL-6*, interleukin 6; *iNOS*, nitric oxide synthase; *I.P.*, intraperitoneal injection; *LBD*, Lewy Body dementia; *LPA*, lysophosphatidic acid receptor; *LPO*, lipid peroxidation; *MBP*, myelin basic protein; *MDA*, malondialdehyde; Mfn1, mitofusin 1; *Mfn2*, mitofusin 2; *mGlur*, metabotropic glutamate receptor; *mHTT*, mutant huntingtin protein; MPTP, 1-Methyl-4-phenyl-1,2,3,6-tetrahydropyridine; *mTOR*, mammalian target of rapamycin; *NA*, not available; *NeuroD1*, neuronal differentiation 1; *NF-kB*, nuclear factor kB; *NGF*, nerve growth factor; *NMDAr*, N-methyl-D-aspartate receptor; *NPCs*, neuronal progenitor cells; *NSC*, neuronal stem cells; *NSE*, neuron-specific enolase; *Nurr-1*, nuclear receptor-related transcription actor 1; *Nyap*, neuronal tyrosine-phosphorylated phosphoinositide-3-kinase; *Olig-2m* oligodendrocyte transcription factor; *Opa1*, optic atrophy 1; *P65*, transcription factor p65; *p70S6*, ribosomal protein p65; *p-AMPK*, phosphorylated AMP-activated protein kinase; *PARP-1*, poly [ADP-ribose] polymerase 1; *Pax6*, paired box protein pax-6; *pCREB*, phosphorylated cAMP response element-binding protein; *PD*, Parkinson’s Disease; *phospho-NF-kB*, phosphorylated nuclear factor kB; *Pitx-3*, pituitary homeobox 3; *p-mTOR*, phosphorylated mammalian target of rapamycin; *PS1*, mutant human PS1 (PS1ΔE9; *PSD95*, postsynaptic density protein 95; *ROS*, reactive oxygen species; *S6K*, s6 kinase; *SA-β-gal*, senescence-associated β-galactosidase; *SD*, Sprague Dawley; *SGZ*, subgranular zone; *SIRT1*, sirtuin1; *SNCA*, synuclein alpha; *SOD*, superoxide dismutase; Sox2, sex determining regions Y-box 2; *Sparc*, secreted protein acidic and rich in cysteine; *Stat3*, signal transducer and activator of transcription 3; *SVZ*, subventricular zone; *Tgm2*, transglutaminase 2; *TH*, tyrosine hydroxylase; *TNF-α*, tumor necrosis factor α; *TrkA*, tropomyosin receptor kinase A; *TrkB*, topomyosin receptor kinase B; *6-OHDA*, 6-hydroxydopamine; ↑, increase; ↓, decrease

A variety of bioactive compounds have been extensively studied due to their neurogenic and neuroprotective potential in animal models of neurodegenerative diseases [210]. These are naturally occurring substances found in different plant products [211]. One of their advantages over conventional drugs is their natural origin which makes them safer [212], but what has sparked more interest was the discovery of their ability to tackle neurodegeneration by multiple mechanisms of action, such as reduction of oxidative stress, neuroinflammation, and inhibition of apoptosis [213]. For example, resveratrol, curcumin, retinoic acid, ginsenoside, and gintonin are some of the bioactive compounds that share these properties and have been widely explored for their potential to alleviate symptoms of diseases marked by decreased neurogenesis [178, 182, 213–215]. These antioxidants can reduce oxidative stress either by scavenging oxidants [185, 188, 215–217] or by activating the transcription factors Nrf2 [218, 219] and HO-1 [219] which are regulators of oxidation. By reducing oxidation levels, there is an increase in the expression of neurotrophins and the survival of NSCs, which allows neurogenesis to proceed smoothly [165]. But these substances can also suppress inflammation by modulating different signaling pathways. For example, the main anti-inflammatory mechanism of resveratrol is its ability to activate the SIRT1/CREB/BDNF signaling pathway [178, 220, 221], which can promote the neuronal survival by inhibiting the activation of microglia and astrocytes [221]. These cells promote neuroinflammation through the release of pro-inflammatory cytokines and chemokines that, in turn, activate more immune cells and lead to a self-perpetuating cycle of inflammation that can cause neuronal damage and death [182, 222]. Another way that these compounds exert their pro-neurogenic potential is through their ability to inhibit apoptosis since they have been shown to modulate apoptotic-related signaling pathways, such as the AKT/mTOR [185]. Substances like curcumin, oleonic acid, and butylphthalide have been shown to activate Wnt/β-catenin [181], Wnt/GSK3β/β-catenin [189], and PI3/AKT [192] signaling pathways, respectively, which are involved in promoting NSC proliferation and survival, and the differentiation of neurons.

Besides these properties, another advantage of bioactive compounds is their high target specificity [223, 224]. For example, multiple studies reported that gintonin has a high target specificity towards lysophosphatidic acid (LPA) receptors - particularly LPA1 and LPA2 receptors [187] – even higher than other known LPA receptor agonists, such as LPA itself [225]. This suggests that gintonin may be a promising strategy to promote neurogenesis, since LPA receptor activation regulates the balance between NSC proliferation and differentiation [226, 227] and has been implicated in the regulation of tau phosphorylation, a key event in the development of AD [228].

One of the shortcomings of these compounds is their low bioavailability [229, 230]. This may have contributed to the limited efficacy of curcumin previously reported [180]. However, recent studies have shown that this issue can be overcome by encapsulating bioactive compounds in functionalized nanoparticles. Indeed, curcumin-loaded nanoparticles significantly increased NSC proliferation and neuronal differentiation in both the hippocampus and SVZ compared to uncoated bulk curcumin [181]. Similarly, in a mouse model of PD (induced with 1-methyl-4-phenyl-1,2,3,6-tetrahydropyridine, MPTP) treated with retinoic acid-nanoparticles was found to be more effective in promoting neurogenesis compared to solubilized retinoic acid [183]. Finally, it is noteworthy that some studies listed in Table 1 reported a contribution of bioactive compounds on neurogenesis only when applied to the pathologic animal models. For instance, the application of rosmarinic and ursolic acid has only promoted neurogenesis in the presence of the neurodegenerative disease, whereas they had no effect when applied in healthy subjects [190]. Some studies also reported that bioactive compounds demonstrated more pro-neurogenic potential than conventional drugs commonly used for the treatment of neurodegenerative diseases, such as donepezil and memantine [190]. Besides donepezil, other synthetic drugs developed to treat specific diseases rather than neurodegeneration presented promising results in promoting neurogenesis as well; these include antidepressants and diabetes medication, which demonstrate pro-neurogenic potential along with their primary therapeutic effects [194].

Evidence reported in Table 1 suggests that many antidepressants target molecular hallmarks of neurodegenerative diseases. Specifically, they have shown that antidepressants can reduce the accumulation of mutant huntingtin (mHTT) [199], α-synuclein [198], and Aβ [196], in animal models of HD, LBD, and AD, respectively, playing a pivotal role in the progression of neurodegeneration [231–233]. While these studies provided limited insight into the precise mechanisms by which antidepressants may reduce the pathogenic burden of the aforementioned proteins, they did reveal a decrease in neuroinflammation [200], an increase in neurotrophins [199], and the activation of BDNF/TrkB [199] and Wnt [196, 200] signaling pathways which, altogether, may have helped reduce their accumulation and consequently, recovery of neurogenesis. In particular, the continued release of pro-inflammatory cytokines from microglia exacerbates neuroinflammation and contributes to this buildup [234–236]. Metformin, an antidiabetic drug, also presented anti-inflammatory and pro-neurogenic results in animal models of PD and AD [194, 206, 207]. In a mice model of AD, metformin protected neurons against apoptotic cell death, and increased neuronal viability; however, this effect was blocked when adenosine monophosphate-activated protein kinase (AMPK) activity was inhibited, suggesting that the pro-neurogenic action of metformin is dependent of AMPK [207], a key enzyme involved in cellular energy regulation and a common target of metformin to tackle diabetes-2 [237]. The authors suggested that hippocampal AMPK activation inhibited mammalian target rapamycin (mTOR) activity, by inhibiting its downstream target, p70S6 kinase [207]. In AD, mTOR enhances Aβ deposition, while AMPK activation decreases mTOR signaling to facilitate autophagy and promote lysosomal degradation of Aβ [238–240]. Other studies have reported that AMPK regulates neuroinflammation and reduces oxidative stress by inhibiting the nuclear factor kappa B (NF-kB), which suggests that the important role of AMPK in neuroprotection may not be only restricted to AD, but also other neurodegenerative diseases [241, 242]. In short, the endeavor to uncover the impact of metformin on neurogenesis has yielded compelling evidence implicating AMPK as a critical mediator in this process, and therefore, it should be regarded as a paramount molecular target to promote neuronal regeneration [243, 244].

Although both antidepressants and metformin provided striking results in stimulating neurogenesis, Mendonça et al. found that the most favorable results in animal models of PD were obtained through the concurrent administration of fluoxetine (Prozac) and metformin [194]. Consequently, it is crucial not to disregard alternative combinatory strategies, as they may hold greater potential.

Small molecules, such as NNI-362 and P7C3-S243, act by modulating specific signaling pathways within the brain that are involved in regulating the growth and survival of neurons [201, 202]. Even though P7C3-S243 did not reduce the pathological features of AD, it improved the behavior of neurologically impaired rats [201]. Additionally, NNI-362 has also emerged as a promising pharmacological agent for promoting neurogenesis. NNI-362 works as a p70S6 kinase stimulator [202] which, as mentioned earlier, is a downstream substrate of mTOR and can stimulate this pathway. While there is some evidence to suggest that the overactivation of mTOR may contribute to AD pathology, its activation can also have positive effects on neurogenesis, since it is also involved in regulating a wide range of cell activities, such as cell growth, proliferation, apoptosis, and autophagy [202, 245]. Indeed, NNI-362 stimulated the phosphorylation of p70S6, which promoted NSC proliferation and differentiation, which ultimately resulted in the reversal of cognitive deficits in aged mice [202]. Of note, other small molecules, such as WAY-316606 proved its pro-neurogenic potential in homeostatic conditions by inhibiting SFRP1 function, which is crucial for the activation of Wnt and Notch pathways and the subsequent activation of neural progenitor cells [246], which may also hold promise to alleviate the symptoms of neurodegenerative diseases.

The role of glutamate receptors in neurogenesis is complex and thus, their precise role in neurodegenerative conditions is not fully elucidated. Despite this, several studies have demonstrated that their inhibition (particularly NMDA [203, 204] and Group II metabotropic glutamate receptors (mGlur) [205]) can increase neurogenesis, suggesting that glutamatergic signaling negatively regulates the process of generating new neurons [203]. The results provided in Table 1 indicate that glutamate antagonists improved the cellular and behavioral function of animal models of neurodegenerative diseases. Particularly, dizocilpine seems to modulate neurogenesis through the activation of the Wnt/β-catenin signaling pathway [203]. However, this finding alone does not fully address the question of how controlling glutamate receptors modulates neurogenesis, and, therefore, further research is required to shed more light on this topic.

Finally, alternative pharmacological approaches have been used to indirectly stimulate neurogenesis. Rather than directly targeting neurons or their progenitors, studies such as those provided by Briyal and colleagues, have been aiming to manipulate other factors that can potentially impact the process of neurogenesis [208, 247]. One such approach involves the modulation of angiogenesis. Drugs like sovateltide (IRL-1620, SPI-1620, or PMZ-1620) have been used for this purpose since it induces both vascular and neuronal modeling [247]. Sovateltide is an endothelin B receptor agonist that has been previously reported to have anti-apoptotic activity [248], increase cerebral blood flow [249], and increase neurovascular repair and remodeling or neuroregeneration, particularly in the SVZ [250]. The activation of these receptors, which are expressed in endothelial, neuronal, and glial cells in the central nervous system [247], results in increased angiogenesis and other neurovascular growth factors in adult NSCs niches, leading to enhanced proliferation and migration of new neurons [247].

Overall, by a brief analysis of Table 1, it becomes clear that there are far more studies focused on promoting neurogenesis in the SGZ of animal models with neurodegenerative diseases, in comparison to the SVZ. It is reasonable to focus on the hippocampus since neurogenesis within this area is critical for learning and memory, which are impacted by neurodegenerative diseases [251]. Nonetheless, in order to grasp the full potential of pro-neurogenic therapies, it is imperative that future research also direct their attention toward the SVZ and other niches, which also possess the ability to regenerate the NSC population [252].

#### Hormone Therapy

Hormone therapy has also been proposed as a possible strategy to promote neurogenesis [253, 254], since hormones — including gonadal hormones, glucocorticoids, and specific metabolic hormones [255] — influence different aspects of neurogenesis, such as proliferation and/or survival of new neurons [81, 256]. This new therapy has emerged as a result of recent findings of hormone dysregulation in neurodegenerative diseases, such as HD [257], AD [258], and PD [259].

An early study has found androgen receptors distributed in many brain areas, especially in the hippocampus and amygdala [260]. At the cellular level, they were found on axons and dendrites, suggesting that androgens (testosterone, dihydrotestosterone, and dehydroepiandrosterone) may have an essential role in neuronal function [261, 262]. Indeed, it has been proposed that in adult males (but not females [263]) androgens enhance hippocampal neurogenesis through the promotion of neuron survival [264]. Specifically, androgens bind to androgen receptors in the CA3 region, which subsequently triggers the expression of survival factors that are retrogradely transported to the newborn neurons in the DG, ultimately promoting their survival and maturation [264, 265]. Despite this, there is limited evidence for the positive effects of androgens in the neurogenesis of experimental models of neurodegenerative diseases. In fact, testosterone presented a limited effect in rescuing neurogenesis in an animal model of HD [257].

A pro-neurogenic potential has also been attributed to a wide variety of female reproductive hormones, including estrogen, progesterone, and prolactin [256]. For example, estrogens (estrone, 17β-estradiol, and estriol) have been shown to play a crucial role in regulating the balance between proliferation and differentiation of NSCs through estrogen-dependent signaling pathways [266], including the MAPK/ERK pathway, which is associated with an increase in neuronal survival [267]. This group of hormones also acts as antioxidants, anti-apoptotic, and induces the expression of growth factors, influencing the neurogenic processes [266, 268]. Finally, they have also been reported to modulate spines and synapse formation which is necessary for the survival of new neurons [269]. This might explain why women who experience premature menopause and do not receive estrogen treatment are at a higher risk of developing AD [270]. Indeed, 17β-estradiol treatment during the early stages of AD pathology in female mice increased the levels of markers of NSC proliferation (BrdU) and mature neurons (NeuN) in the hippocampus, which was supported by the recovery of cognitive function [271].

Other hormones related to reproductive health have also presented multifaceted neuroprotective and neurodegenerative processes, including progesterone [272], its metabolite allopregnanolone [273, 274], and progestin [275, 276]. Progesterone exerts its neural effects through multiple signaling pathways, which include binding to specific progesterone receptors that regulate gene expression [267]. In particular, membrane progesterone receptor β (mPRβ/Paqr8) promotes neurite outgrowth via extracellular signal-regulated kinase (ERK) phosphorylation [277]. Some of the pro-neurogenic effects of progesterone are partially mediated by its neuroactive metabolites, including allopregnanolone [78]. Allopregnanolone has a high affinity for GABA_A_ receptors, specifically the GABA-chloride channel complex, which induces membrane depolarization upon activation. This ultimately leads to the activation of kinases that regulate the expression of genes and proteins involved in the cell cycle of NSCs, promoting their regeneration [278]. Despite this, a phase 3 clinical research trial showed a 100% failure rate for progesterone as a treatment for traumatic brain injury [279]. But, because traumatic brain injury is a very heterogeneous and complex disorder [280], these results should not dismiss the potential that progesterone has previously shown [281]. Alternatively, the pro-neurogenic potential of some progestins, synthetic analogs of progesterone with a similar mode action [280], have been thoroughly investigated, with Nestorone receiving particular attention due to its high selectivity for progesterone receptors, greater than progesterone itself [275, 280].

Naturally occurring or synthetic estrogens and progesterone are not the only hormones capable of stimulating neuronal proliferation. Prolactin has emerged as another hormone with pro-neurogenic effects based on findings indicating increased NSCs in the SVZ of female mice during pregnancy and lactation [282]. Further, administration of a prolactin analog (palm^11^-PrP31) resulted in elevated neurogenesis in the hippocampus of male mice models of AD [283]. While the evidence is promising, further research is required to fully comprehend the potential of prolactin as a strategy to promote neurogenesis [284, 285].

It is worth noting that some of the reported studies proved that these hormones can only promote the innate regenerative capability of a pathological brain during the early to mid-stages of the disease [286]. Additionally, hormone therapy to stimulate neurogenesis remains controversial, not only because it is a complex medical intervention but also because different studies have reported stimulatory and inhibitory effects [287, 288]. This may be attributed to the complex influence of different factors in hormones, such as gender, age, genetics, and environmental influences [81].

#### Gene-Based Therapies

**Fig. 4:**
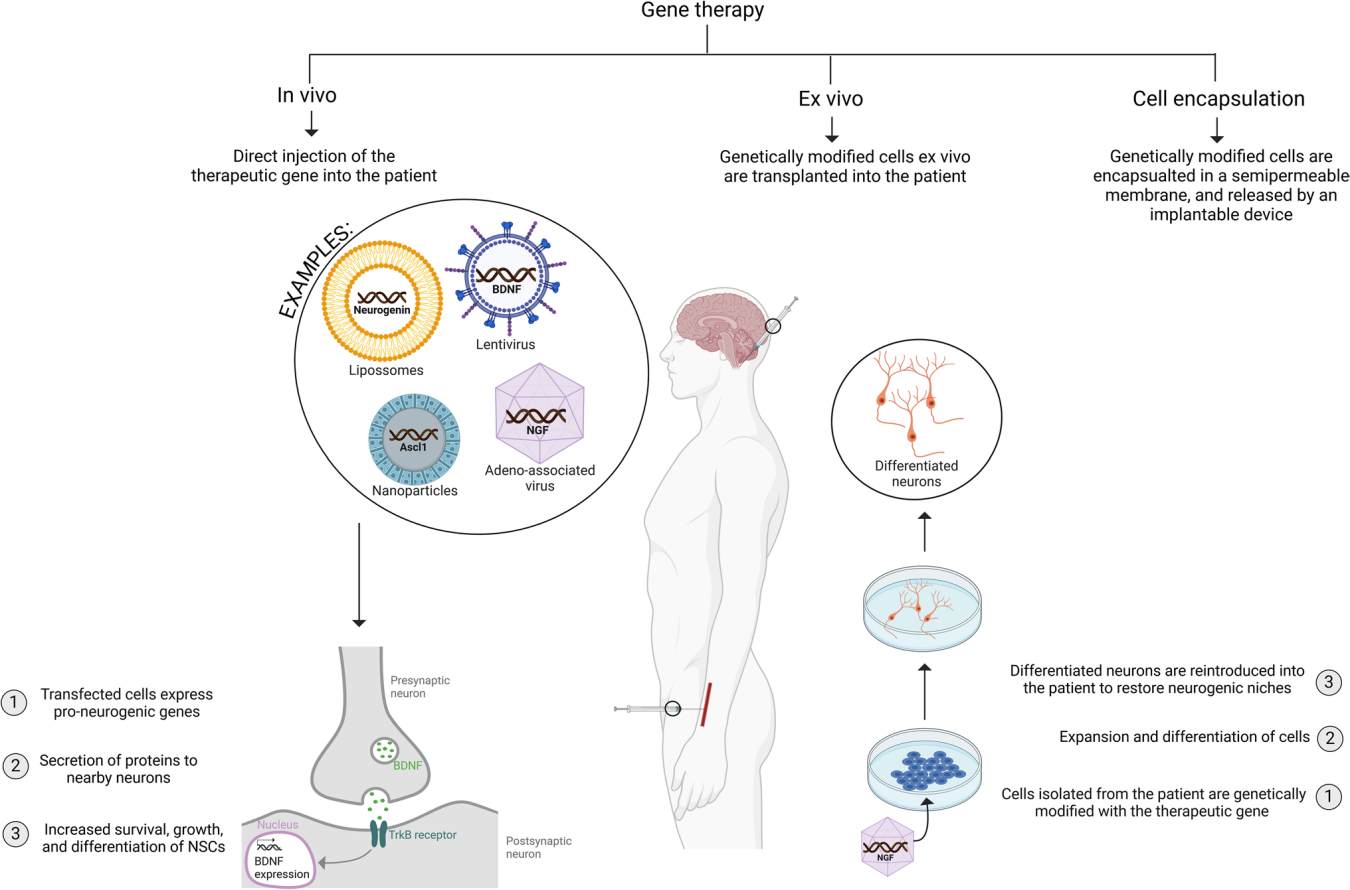
Gene-based pro-neurogenic therapies. Through different viral and non-viral gene carriers, therapeutic genes may be inserted into the patient, where they will selectively target neurons or their progenitors’ cells, transfect them, and integrate the gene of interest onto the cells’ genome, to promote their survival, growth, or differentiation. Another approach is to induce neurogenesis outside the organism. In the ex vivo strategy, cells may be transfected with the therapeutic gene, which will induce their differentiation. Once differentiated, cells can be reinserted into the targeted niche, where they will either repopulate the damaged neurogenic niche, or stimulate the differentiation of surrounding cells. BDNF, brain derived neurotrophic factor; NGF, nerve growth factor. Created with Biorender.com

Gene-based therapies, which are based on genome manipulation, have emerged as a way to promote neurogenesis by the modulation of gene expression in NSCs and other cell types. This can be performed through a repertoire of gene-manipulation tools currently available such as viral and non-viral delivery strategies (nanoparticles, ribonucleoproteins, electroporation, etc.) [289–291]. These strategies enable the introduction of therapeutic genes into the target cells, either in vivo or ex vivo, each offering distinct mechanisms for promoting neurogenesis [292] (Fig. 4).

In the in vivo approach, innate neurogenesis is stimulated through the injection (localized or systemic delivery routes) of the aforementioned gene-manipulation tools into the organism, which allows the therapeutic genes to be delivered and expressed within the target neurogenic niches [293]. These genes can then enter NSCs, where they modulate gene expression, promoting their proliferation, survival, or differentiation into neurons [294]. These include genes (most commonly neurotrophins [295, 296]) that control cell fate decisions, including the Trk, Wnt, and bone morphogenetic protein (BMP) signaling pathways [297, 298]. Other genes have been studied for their potential of regulating neurogenesis, such as BRI2 [299, 300], Neurogenin 1 and 2 [301, 302], Ascl1 [303], among others [294], which may also hold promise in gene therapy applications.

Other therapeutic approaches based on gene delivery were already successfully applied in the treatment of several neurodegenerative diseases, including AD, PD, and HD [304]. For example, BDNF delivery by injections of recombinant adeno-associated virus enhanced the recruitment of progenitor cells to the lesioned areas of adult rat brains and promoted neuronal differentiation [305]. Similarly, a single injection of adenoviral BDNF also increased the recruitment of new neurons to the OB and striatum of adult rats [296]. Moreover, induction of striatal neurogenesis by adenoviral-mediated overexpression of BDNF, correlated with delayed motor impairment, and improved survival in a murine model of HD [306]. Viral-induced overexpression of NGF was reported to promote neurogenesis in two different rodent models of ischemic brain injury [307]. Furthermore, IGF-I gene therapy using an adenoviral vector increased the number of immature neurons in the DG of aging rats [308].

The manipulation of the expression of these genes can have implications in the treatment of neurodegenerative diseases, but there are still some limitations. In a clinical trial for PD, intrastriatal infusion of an adenoviral vector was associated with an increased risk of intracranial hemorrhage and headaches [309]. Additionally, permanent genetic modification of the patients’ brain cells, coupled with the inability to control or interrupt the release of the bioactive substance raises safety and regulatory concerns [310].

Thus, future advances in delivery methods could improve the clinical significance and therapeutic outcomes of gene therapy. The ex vivo strategy is another promising alternative, in which cells can be manipulated and differentiated outside the organism before being reintroduced into the patient [311]. In this way, researchers can potentially generate large numbers of specific types of neurons for transplantation (or just research purposes). In other words, they can induce exogenous neurogenesis.

It is worth noting that there have been major advances in many other technologies. For example, genetically modified encapsulated cells were already tested in both animals and humans, showing promising results in different research fields [310, 312]. In a clinical trial patients with mild to moderate symptoms of AD, cell encapsulation biodelivery of NGF to the basal forebrain proved to be safe and increase cognition [310, 312]. This technology comprises the use of cells, which are genetically modified to secrete a therapeutic compound, and then are encapsulated before being delivered into the patient [313]. This technology has many advantages over the traditional in vivo and ex vivo gene therapy approaches, namely, the targeted delivery of the compound of interest, whose release can also be controlled using an implantable and retrievable medical device [314]. Additionally, capsules can be engineered to protect the cells from the host’s immune system [310, 314].

### Exogenous Neurogenesis Induction

Although some studies have suggested that neurogenesis is enhanced in certain regions of the brain in response to neurodegenerative diseases (potentially as a compensatory mechanism) [315], typically, in the later stages of these illnesses, the innate neurogenic capacity of the brain is limited [315]. Therefore, stimulating the production of new neurons may not be enough to combat the extensive neuronal cell loss that occurs in all brain regions (including non-neurogenic areas) during the aging process and neurodegenerative diseases [30]. In these cases where the existing damage is far too extensive, replacing the cells that are lost or damaged, may be a therapeutic option [316].

#### Cell-Based Therapies

Stem cell therapy, also known as regenerative therapy, typically focuses either on cellular replacement or on providing trophic support to damaged or dysfunctional tissues [317]. This strategy may improve neurogenesis by introducing new stem cells into specific regions of the brain, that then differentiate into neurons [316]. Different types of stem cells have been studied for transplantation purposes, including embryonic stem cells [318], fetal stem cells [319], adult stem cells such as NSCs [320], induced pluripotent stem cells (iPSCs) [321], and even mesenchymal stem cells (MSCs) [322].

Totipotent embryonic stem cells have proved to reverse cognitive deficits after transplantation into the frontal region of the cortex of a mouse model of AD, induced through lesions on the nucleus basalis Meynert, which is a brain region vulnerable to neurodegeneration [323]. These cells successfully differentiated into cholinergic and serotonin-positive neurons, the loss of which has been linked to the manifestation of AD symptoms [323]. However, besides being associated with a risk of tumor formation, this approach also faces ethical and legal issues in the clinic, limiting their study and application [324]. Fetal stem cells face fewer ethical and legal problems since they are obtained from fetal tissues that would otherwise be discarded [325]. However, they are more difficult to obtain and far more restricted in their ability to differentiate into specialized cell types, which has been limiting their application in regenerative medicine [325]. Nevertheless, a phase 1 clinical trial employed their transplantation in individuals with progressive multiple sclerosis and has yielded promising results [319], underscoring the potential of these cells in treating other neurodegenerative disorders.

NSCs transplantation has also proved to differentiate into neurons after engraftment [326], improving cognitive scores in animal models of neurodegenerative diseases [327]. However, their transplantation goes beyond their ability to replace lost cells, since they also have the potential to serve as delivery vehicles of therapeutic genes [328], to secrete growth factors and other molecules that can promote the survival and growth of existing neurons and provide cues to stimulate the production of new ones [316]. In other words, this trophic support creates a more favorable environment for neural repair and regeneration [329]. Other studies even suggest that they have an anti-inflammatory role to protect the brain from cerebral inflammation [330]. Alterations in neuroinflammation status following stem cells’ transplantation might create a microenvironment conducive to enhanced neurogenesis [331].

NSCs for transplantation purposes can be obtained from brain tissue [332], may result from the differentiation of the aforementioned cells (embryonic and fetal stem cells [333]) or alternatively, they may result from the reprogramming of somatic cells, resulting in iPSCs [334]. NSCs derived from iPSCs have the advantage of being generated from the patient’s own cells, which reduces the risk of immune rejection and eliminates the need for immunosuppressive drugs [334]. This strategy holds promise for the creation of patient-specific NSCs, which provides a more personalized approach [335]. However, one of the limitations of this procedure is related to the heterogeneity of these cells (i.e., differences in their gene expression profiles, and varying degrees of maturity), meaning that when they are implanted into the brain, not all of them may successfully differentiate into the intended type of mature and functional neurons since they may adopt alternative cell fates. Research such as the one conducted by Xu et al. is crucial to tackle this issue since it may help to provide a better understanding of cellular heterogeneity throughout the differentiation process while introducing a method based on surface markers identification to accurately separate the right cells from the unwanted ones [336].

Despite these shortcomings, the potential of NSCs’ transplantation has already been supported by the successful results of many pre-clinical and clinical studies [337, 338]. For example, induced NSCs that were converted from mouse fibroblasts and transplanted into the striatum of a PD mouse model were able to migrate to the damaged *substantia nigra* and differentiate into dopaminergic neurons, which enhanced functional recovery [339]. Recently, Schweitzer *et al*. also documented a successful procedure involving the implantation of midbrain dopaminergic progenitor cells derived from the patient's own iPSCs. This approach was conducted in a patient diagnosed with idiopathic PD, resulting in remarkable improvements in clinical symptoms observed 18-24 months post-implantation. Such outcomes further highlight the potential of iPSC-based strategies in addressing neurodegenerative conditions like PD [338].

Another interesting approach based on cell reprogramming is glia-to-neuron reprogramming, which was first reported 20 years ago [340], but only recently has it been receiving more attention [341]. This technique aims to take advantage of the regenerative ability of resident glial cells [341] to directly convert them into neurons through cytokines, epigenetic factors, and transcription factors [342, 343]. Unlike the method previously described, in which cells must be reprogrammed in vitro before transplantation, glia-to-neuron conversion takes place directly in the brain [344], and was already reported to occur spontaneously as a response to specific brain trauma [345, 346]. However, inducing this reprogramming remains a major challenge, and ongoing research efforts are focused on refining the technology. Another crucial aspect is whether the newly converted neurons can successfully integrate into existing neuronal circuits and perform their intended functions [347]. While this remains uncertain, the ultimate goal is to create a technology that can replenish damaged areas with healthy neurons, as well as potentially reduce gliogenesis, which can act as a protective mechanism to minimize and repair brain injuries that, under specific circumstances, can lead to harmful effects [348].

Finally, MSCs have also demonstrated the ability to transdifferentiate into functional neurons [322, 349]. But beyond their role in cellular replacement, MSCs can also help treat neurodegenerative disorders through the expression of neurotrophic factors such as BDNF, NGF, and IGF-1 [331, 350, 351].

Even though stem cell therapy has shown remarkable results in improving cognitive deficits and neuronal loss in neurodegenerative diseases, the underlying mechanisms are not yet fully understood. Additionally, it is necessary to further understand how to create a microenvironment capable of sustaining and functionally integrating grafted and/or reprogrammed cells [317]. Nonetheless, regenerative stem cell therapy is an enticing therapeutic strategy for the retardation of neuronal loss, recovery of endogenous neurogenesis, and improvement of cognitive functions in neurodegenerative diseases.

## Clinical Trials

As seen throughout the present study, several pre-clinical studies in animal models of neurodegenerative diseases have explored different strategies to enhance adult neurogenesis, some with promising results; however, clinical trials in this area are still relatively limited, as summarized in Table 2.

**Table 2:** Clinical trials of therapies to promote adult neurogenesis of patients with neurodegenerative diseases

Pro-neurogenic therapeutical intervention	Condition or disease	Clinical trial Identifier	Clinical trial Phase	Start date	Completion date	Status	Results	Ref.
Exercise and tDCS	Major and mild neurocognitive disorder due to AD or mixed AD/vascular disease	NCT03670615	N/A	2018	2025	Recruiting	Not posted	NA
Dietary restriction and/or extended periods of mastication	Aging and cognitive decline	NCT03457870	N/A	2018	2020	Completed	↑ Recognition memory, and pattern separation	[352, 353]
Intravenous administration of sovateltide	AD, dementia	NCT04052737	II	2019	2023	Completed	Not posted	NA
Intravenous administration of allopregnanolone	AD, late onset AD	NCT04838301	II	2023	2025	Not yet recruiting	Not posted	NA

Abbreviations**:** *AD*, Alzheimer’s disease; *AAV2*, adeno-associated virus type 2; *N/A*, Not Applicable; *tDCS*, transcranial direct current stimulation

One reason for this could be the difficulty in translating these findings to clinical settings [354]. Additionally, the lack of non-invasive techniques to directly measure neurogenesis in live humans may also contribute to this challenge [355]. Currently, there are only indirect methods to estimate neurogenesis in live humans. These include neuroimaging techniques such as magnetic resonance imaging (MRI) [356], biomarkers in blood [357, 358], changes in cognitive function or behavior [359], and radiolabeling [360]. However, these lack resolution, sensitivity, and specificity, since they fail to detect the exact changes in the number or activity of new neurons, to differentiate between cells, and are unable to study the process in real time [355, 359].

Regarding neurostimulation, there is currently only one clinical trial in the recruiting phase (NCT03670615), aiming to assess how exercise combined with tDCS (20 min sessions at 2 mA, 5 times per week for 2 weeks applied through 35 cm^2^ bitemporal electrodes) affect the cognition and brain plasticity of patients with major and mild neurocognitive disorder due to AD or mixed AD/vascular disease [357]. In this study, changes in neurogenesis will be assessed through biomarkers (BDNF), obtained through blood work [357].

Although animal studies have clearly demonstrated the link between physical exercise and increased neurogenesis [361], human studies have provided mixed results [362]. To date, no clinical research has specifically targeted the effect of exercise on neurogenesis. Still, some studies have found that physical activity can enhance cognitive performance in patients with PD [363, 364], while others have observed increases in hippocampal volumes [365]. It remains unclear, however, whether these outcomes are a consequence of increased neurogenesis. Similarly, a different clinical study found that mastication, which can be considered a mild form of exercise [353, 366], along with intermittent calorie restriction, benefited hippocampus-dependent cognition in older individuals [352, 353]. Another study (NCT03457870) aimed at assessing the effects of short-term intermittent and continuous calorie restriction on insulin sensitivity in obese individuals, and also found a memory improvement linked to hippocampal neurogenesis [355]. But these studies present some limitations that should be addressed in the future. For instance, since poor oral health appears to influence cognitive function [367, 368], this factor should be considered in upcoming clinical studies. But most importantly, these studies did not address cellular and molecular mechanisms, particularly neurogenesis-associated markers, which inhibits to conclude with certainty that these improvements were a direct result of neurogenesis.

An extensive literature search allowed to conclude that there are currently no ongoing clinical trials that have specifically assessed whether bioactive compounds can promote adult neurogenesis. However, a meta-analysis of 225 patients showed that resveratrol has the potential to enhance mood but with no significant impact on factors related to memory and cognitive performance [369]. On the other hand, curcumin can improve the cognition of healthy and non-demented adults but has detrimental effects on patients with AD, indicating selective effects on different regions of the brain and cognitive domains [370, 371]. It is recommended that upcoming clinical trials with bioactive compounds improve their bioavailability by using innovative approaches such as nanoformulations or in combination with metabolism inhibitors, which should allow them to remain active in the body for longer periods [369].

Sovateltide is the only pharmaceutical candidate targeting adult neurogenesis that has undergone a clinical trial for the treatment of AD and dementia (NCT 04052737). As previously reported in Table 1, pre-clinical studies with this drug have shown that it is involved in neuronal cell survival and the restoration of adult neurogenesis in neurodegenerative diseases [208, 247]. Based on these results, sovateltide was recently on a phase 2 clinical trial and upcoming results should provide information about whether it augments the activity of NSCs in the brain.

Consistent with animal studies, intravenous administration of allopregnanolone was well tolerated and safe across all doses in people with early AD [372]. Therefore, this hormone therapy soon will enter a phase 2 efficacy trial to determine if this therapy can restore structural integrity and cognitive function in patients with AD [373]. Preliminary results from MRI indicate that allopregnanolone administration reduces (and sometimes reverses) hippocampal volume, which was augmented in patients with mild AD. Additionally, this strategy successfully strengthened local, inter-regional, and network level functional connectivity in brain regions vulnerable to AD pathology, which supported advancement to a phase 2 clinical trial (NCT04838301) [373], in which changes in biomarkers of neurogenesis will be addressed. Despite these interesting results, no other hormones are currently involved in clinical trials.

Besides the clinical studies included in Table 2, there are many more that have been exploring the potential of inducing neurogenesis to alleviate symptoms and treat a range of neurological disorders, with promising results. These include conditions such as depressive and bipolar disorders, schizophrenia, traumatic brain injury, and many others (e.g.: NCT03608462, NCT05755321, NCT01552837, NCT03345550, etc.), which were not included since it goes beyond the scope of the current review. It is worth noting that the plethora of research exploring neurogenesis demonstrates its potential as a promising area for further investigation into neurodegenerative diseases. However, remain several limitations to this therapeutic approach that require further attention. One of the main challenges is that many clinical studies lack follow-up assessments, which are necessary to determine the acute effects of interventions on neurogenesis and, consequently, memory and cognition. Furthermore, the lack of non-invasive methods for measuring adult neurogenesis in live humans is currently the biggest obstacle facing this field [355, 374]. Without accurate measures, it is challenging to translate clinical research into clinical practice. As such, it is necessary to develop appropriate in vivo markers that can precisely measure neurogenesis.

## Conclusions and Future Perspectives

Based on the available evidence, it appears that it is possible to manipulate neurogenic niches to stimulate neurogenesis in the adult brain, potentially offering a promising treatment strategy for neurodegenerative diseases and other neurological disorders. The activation of endogenous neural stem/precursor cells in response to various external stimuli, including electrical and magnetic stimulation, physical exercise, environment enrichment, diet changes, and pharmacological interventions, suggests that these approaches could offer a generally safe way to promote neurogenesis in adults. However, the effect of these strategies may not be as pronounced as those achieved through more invasive therapies such as stem cell transplantation, which may produce rapid and significant changes in the brain. Nonetheless, these invasive therapies are still experimental and may carry significant risks and side effects.

With this work, it became clear that stimulating neurogenesis in animal models of neurodegenerative diseases presents a great challenge. This challenge primarily stems from the complexity and multifactorial nature of neurodegenerative diseases, which involve various pathological processes that extend beyond impaired neurogenesis. Despite this, most studies still focus on promoting neurogenesis in animal models of neurodegenerative diseases, as a way to mitigate existing symptoms rather than preventing the onset of dementias. To enhance the understanding of neurogenesis as a therapeutic tool, researchers should take a step back and shift their focus towards investigating the potential of promoting neurogenesis in healthy subjects. Possibly, by stimulating neurogenesis prior to the onset of the disease, we might be able to preserve the plasticity of brain circuits, and thereby counteract the progressive neuronal loss, and cognitive decline characteristic of neurodegenerative diseases.

One final remark, while some rodent studies have shown promising results, it is difficult to extrapolate these findings to humans since these two mammals have major differences in brain size, organization, and function. This indicates a need to search for and develop alternative models to better mimic the molecular and cellular mechanisms of human neurogenesis. Additionally, although clinical trials are trying to make it to clinical practice, the lack of reliable biomarkers and standardized techniques to measure neurogenesis in humans makes it even more challenging. Finally, ethical considerations and regulatory requirements hinder large-scale clinical trials in humans, particularly when experimental treatments involve invasive procedures or carry potential risks.

Therefore, although clinical trials are a crucial step in determining the clinical viability of the discussed pro-neurogenic approaches, these limitations are blocking progress in the field, and therefore, they should be addressed soon.

## Data Availability

No datasets were generated or analysed during the current study.
